# Editorial: Comparative neuromechanical circuits of the sensorimotor system

**DOI:** 10.3389/fnint.2022.975948

**Published:** 2022-07-18

**Authors:** T. Richard Nichols, Monica A. Daley

**Affiliations:** ^1^School of Biological Sciences, Georgia Institute of Technology, Atlanta, GA, United States; ^2^Department of Ecology and Evolutionary Biology, School of Biological Sciences, University of California, Irvine, Irvine, CA, United States

**Keywords:** sensorimotor integration, musculoskeletal system, computational model, comparative motor control, central pattern generator (CPG) control, inertial coupling

The musculoskeletal and nervous systems have evolved together to mediate stable and coordinated posture and movement. Although considerable information is available about the structure and function of both systems independently through the research areas of biomechanics and neurophysiology, progress in understanding the mechanisms of motor control has been accelerated by the study of these two systems together, resulting in the emerging area of neuromechanics. This Research Topic includes papers representing this integrated approach to the study of motor function. These contributions also feature a comparative approach, seeking insights from the study of diverse organisms, from coordination in multi-segmented invertebrates to interneuron connectivity in cats, to multi-sensory convergence in human motor control. A wide range of contemporary modeling and experimental approaches are featured, including computer simulations, recording of single motor units and interneurons by micro-electrode arrays, functional imaging, and the use of robotics to control interactions between body and environment. We have grouped the articles into three subtopics, focusing first on the interplay between musculoskeletal mechanics and neural control, then on stretch reflexes and sensorimotor integration in the spinal cord, and finally on the way in which limb mechanics are represented in the human brain and on descending control of spinal pathways.

The first group of articles directly addressed the importance of body and limb mechanics in understanding mechanisms of motor coordination. The first three articles discussed computational models that represent integrated neural and musculoskeletal pathways for repetitive movements. In the first (Ambe et al.), the authors developed a neuromechanical model for multi-legged locomotion. The mechanical model consisted of a number of segments represented by masses interconnected by damped springs. Each segment also included an oscillator with phase-resetting sensory feedback. The authors showed that multiple, naturally observed gait patterns for coordinating multi-legged locomotion emerged from this simple decentralized motor control system. In the second paper (Okamoto et al.), the authors used a simplified neuromechanical model of human walking to investigate the phenomenon of statistical persistence of stride intervals. The model consisted of a simple compass type biomechanical walking model coupled to a Central Pattern Generator (CPG) based controller. The control model included a phase oscillator to generate feedforward motor commands, coupled to phase resetting sensory feedback. The authors found statistical persistence in the model with phase resetting feedback, which is lost in the model without phase resetting feedback. In the third paper (Prilutsky et al.), the authors integrated experimental evidence with a model of the feline hindlimb and spinal cord to understand the paw shake response, a behavioral characterized by high velocities and accelerations. The model consisted of central pattern generating circuits, sensory feedback and a multi-segmented limb with realistic inertial properties of the limb segments. Combining all three components successfully reproduced the whip-like dynamics that are experimentally observed in the paw shake. In the final article in this group (Ludvig et al.), experiments with human participants were used to explore tasks that either used or opposed the natural impedances of the ankle joints. The latter tasks required subjects to modulate muscular activation patterns more than for the former task and were perceived to be more difficult by the subjects. These findings illustrate the importance of leveraging joint impedances to effectively perform natural movements.

The second group of articles addressed structural and functional aspects of sensorimotor integration. Classical studies of feedback from muscles onto motoneurons had shown that motor units concerned with posture and stabilization tend to receive higher densities of input from muscle spindles than those concerned with more dynamic tasks. In an experimental study using human participants (Nicolozakes et al.), the authors showed that the rotator cuff muscles, muscles that are primary stabilizers of the shoulder, exhibit stronger stretch reflexes than the larger prime movers of the shoulder. These results are consistent with the role of stretch reflex to regulate joint stiffness to stabilize the shoulder against disturbances. Investigating functional connectivity within the spinal cord is also critical to understanding sensorimotor integration, because most inputs to spinal motoneurons come from interneurons that integrate sensory and descending inputs, rather than directly from the periphery. In the second study in this group (Zaback et al.), the authors introduced a new method to map the functional connectivity between cutaneous sources and motoneurons. Microelectrode arrays were employed to record single motor units and interneurons in the spinal cord, and connectivity was inferred from correlations between these two sources. The method was validated using pathways that are known to have more or less direct connections to these interneurons. In the third study (Thompson et al.), a comparative approach was used to understand the differences in firing patterns of motor units in response to tendon vibration in cat and man. New, multi-unit recordings of single motor units from muscles were used in both species. The authors found that motor units tend to fire in integral multiples of the vibration frequency in cat but not man. These different firing patterns might be explained by greater temporal dispersion from longer conduction distances in humans, resulting in a smoothed and more uniform synaptic input.

The third group of articles focused on brain and nervous system-wide mechanisms of motor coordination and the convergence of different sensory sources for motor control, continuing the themes of stabilization and impedance control. In a review article (Jayasinghe et al.), the task of stopping a movement is used to introduce a hybrid model of motor control. Continuing the theme of stabilization and impedance control introduced above, the authors argue that there are two independent aspects of motor control, on the one hand, the control of movement trajectory and compensation for inertial coupling between limb segments, and on the other hand, stopping and stabilization of movement. The authors further argued that stabilization is most readily explained by impedance control. Continuing the theme of stabilization, the authors of the second article in this group (Suminski et al.) addressed the contributions of visual and proprioceptive feedback to joint stabilization in response to torque perturbations. When both sources of feedback were congruent, they both contributed; however, when visual information was unreliable, it was apparently ignored. This finding led the authors to reject a long-standing model of multi-sensory convergence. These studies were carried out with a robotic and virtual reality system, as well as fMRI imaging to assess the involvement of principle brain areas. The results of brain imaging provided insights into the neural substrate of the observed interactions between the two sensory sources. Closing the loop on spinal cord and brain mechanisms of motor coordination, the final article (Xu et al.) addressed the influence of brain areas on spinal circuitry by conditioning a measure of the stretch reflex (H- reflex) with transcranial magnetic stimulation (TMS). The cortex influences the spinal cord through both rapid, direct and slower, indirect pathways. By recognizing this, the authors were able to assess the influence of cranial stimulation on the direct and indirect pathways by altering the intervals between cranial and peripheral nerve stimulation. This approach provides a method and normative reference values for investigating these two pathways in health and disease.

## Author contributions

All authors listed have made a substantial, direct, and intellectual contribution to the work and approved it for publication.

## Conflict of interest

The authors declare that the research was conducted in the absence of any commercial or financial relationships that could be construed as a potential conflict of interest.

## Publisher's note

All claims expressed in this article are solely those of the authors and do not necessarily represent those of their affiliated organizations, or those of the publisher, the editors and the reviewers. Any product that may be evaluated in this article, or claim that may be made by its manufacturer, is not guaranteed or endorsed by the publisher.

